# Introducing Open Dialogue as part of the WHO QualityRights Project in South Korea: experiences and opinions from an introductory workshop and 1-year pilot practice

**DOI:** 10.3389/fpsyg.2024.1426122

**Published:** 2024-10-17

**Authors:** Sooni Cho, Yong Hyuk Cho, Jai Sung Noh, Seong Kwon Jeong, Shin Kwon Kim, Seongsu Kim

**Affiliations:** ^1^Eum Hospital, Yong In, Republic of Korea; ^2^Department of Psychiatry, Ajou University School of Medicine, Suwon, Republic of Korea; ^3^Department of Medical Humanities and Social Medicine, Ajou University School of Medicine, Suwon, Republic of Korea; ^4^Dawon Mental Health Clinic, Suwon, Republic of Korea

**Keywords:** Open Dialogue, WHO QualityRights, human rights, recovery practice, person-centered, Open Dialogue training, implementation of Open Dialogue

## Abstract

This study explores the subjective experiences of participants in a 5-day Open Dialogue (OD) workshop and a 1-year pilot practice, conducted as part of the WHO QualityRights Project in South Korea. Twenty-four participants, selected through purposive sampling, completed surveys immediately after the workshop and 1 year later. Data were analyzed through both statistical and thematic approaches. A statistically significant decrease in the availability of “Flexibility and Mobility” was observed across all participants (*p* = 0.044) and a significant reduction in the availability of “Tolerance of Uncertainty” (*p* = 0.04) was noted among participants who engaged in network meetings over the course of 1 year. Qualitative analysis revealed that participants initially felt ambivalent toward OD due to systemic, cultural, and professional challenges. However, through experiential learning, their ambivalence shifted to hope, fostering solidarity and a more positive outlook for future OD practice. Participants recognized that implementing OD supported human rights, while addressing personal, organizational, and policy challenges. The findings provide important insights for developing OD training and implementation guidelines in South Korea. Recommendations include focusing on experiential learning and selecting mixed-group trainees from catchment area institutions, emphasizing the support of client rights, and considering individual, organizational, and systemic levels for successful implementation. This study represents a new case of OD dissemination through a top-down national research and development project and its integration into the WHO QualityRights service package, suggesting complementary potential between OD and global human rights-based mental health initiatives.

## 1 Introduction

Open Dialogue (OD) is a system of mental healthcare developed in Western Lapland, Finland. Two essential ingredients of OD are the therapeutic and philosophical approaches to being with people in times of crisis or need; OD is also a way of organizing mental health services that maximizes the possibility of being able to respond to people (Jackson and Perry, 2015; Putman, 2021b). OD incorporates aspects of individual psychodynamic therapy and systemic family therapy, with a focus on the centrality of relationships and the promotion of connectedness through family and network involvement (WHO, 2021, p. 9).

Over the decades of its evolution, seven key principles of OD (Seikkula et al., 2001) have emerged: (1) immediate help; (2) social network perspective; (3) flexibility and mobility; (4) responsibility; (5) psychological continuity; (6) tolerance of uncertainty; and (7) dialogism. The first five principles are concerned with the structure of the service, and the last two with the form of practice; in reality, all of the principles are interrelated and depend upon each other (Seikkula and Olson, 2003). Therefore, in the effective implementation of OD, practical skills and teamwork are necessarily linked to how service systems are coordinated.

In a 2018 register-based cohort study conducted in Finland, the outcomes of OD were evaluated in comparison with a large nationwide control group covering a timespan of ~19 years. The duration of hospital care, disability allowances, and the need for neuroleptic medication remained significantly lower in the OD cohort (Bergström et al., 2018). Further, it has been noted that OD participants tend to have better employment outcomes than those treated conventionally (Seikkula et al., 2006). Another national 5-year cohort study found that the Western Lapland catchment area had the lowest figures in Finland for the duration of hospital treatment and disability pensions (Kiviniemi, 2014). Qualitative studies also found that people using the service felt positively about it, along with the families and professionals involved (Tribe et al., 2019).

The World Health Organization (WHO) has developed and disseminated the QualityRights initiative, which uses a multi-component framework and strategies to promote mental health systems, services, and practices that prioritize respect for human rights in line with the United Nations (UN) Convention on the Rights of Persons with Disabilities (CRPD) (Funk and Bold, 2020). In the progress of this project, the WHO has listed OD in its guidance on community mental health services, promoting person-centered and rights-based approaches as best practices for mental health crisis response services (WHO, 2021, p. 27). In the context of these impressive achievements, OD has spread across countries and is growing rapidly, with more than 100 centers in 24 countries on five continents offering this approach (Pocobello et al., 2023).

### 1.1 OD: diversity in initial introduction in different contexts

There is still no mental health system outside Western Lapland in Finland, where the seven principles of OD are fully implemented (Buus et al., 2017), likely due to differences in existing service delivery and collaboration systems across countries. Therefore, there has been research on practices and a growing discourse on how OD has been implemented in different countries with different mental health systems (Pocobello et al., 2023).

Despite these differences in context, a common thread in OD adoption across countries is that the first step is to introduce training programs to equip service providers with the skills needed to implement OD. Nowadays, many countries generally introduce foundation training in OD for their service workers, with durations ranging from 16 to 20 days (Putman, 2021a).

However, there are several examples of shorter introductory workshops or short-term trainings as a preliminary step to full-scale training. This may be a viable way to introduce and spread OD when there is still a lack of social consensus for full-scale training and implementation and the necessary time, funds, and policies are lacking.

A study of participants at an OD conference in the UK found that while many agreed with the potential for positive changes in terms of clinical values and teamwork, implementation would require a commitment of resources and a shift in professional attitudes and service culture (Razzaque and Wood, 2015). Meanwhile, a study in Australia found that even participants in a fairly short 42-h OD training and pilot reported that the “different” learning experience they had received changed their perspective on therapeutic approaches and strengthened the bonds among them (Buus et al., 2023). Additionally, the experience of implementing the short training in two public health organizations suggested the need for a shift in organizational culture and leadership to become more relationship-oriented (Lennon et al., 2023). A study of a pilot after a short period of training in a psychiatric inpatient unit in the US suggested that this approach was effective in increasing the efficiency of daily clinical activities, improving patient-provider communication, and creating a more patient-centered care environment (Rosen and Stoklosa, 2016). A study of a group of mental health professionals who experienced only a short OD online workshop with no formal training reported that the dialogical approach of regular supervision over a significant period of time had numerous meaningful impacts on both the participants' clinical practice and their professional teams (Skourteli et al., 2023). Additionally, several short OD workshops of 2, 3, or 4 days have been conducted in various places [Training Course at Yale University | Institute For Dialogic Practice, 2022; Brown, 2023; Dialogue (R)Evolution, 2022], but research on these workshops is relatively scarce compared to that on foundation or full trainings.

In many relevant studies, service providers who participated in an introductory short workshop/training and pilot implementation to introduce OD for the first time indicated that an approach grounded in OD principles required change on many levels, including their professional identity, teamwork within the organization, and collaboration with other sectors; they often mentioned the difficulty of applicability due to the differences between OD practices and traditional services.

Thus, to properly plan the introduction of OD for the first time in a country or system, an introductory phase prior to formal training and implementation requires careful design to minimize conflicts with existing services and subsequent resistance, maximize the experience of the unique strengths of OD training, and ensure that all participants are motivated to change their own clinical practice and organization.

### 1.2 Introduction of OD as part of the WHO QualityRights Project in South Korea

In South Korea, a lack of legislation and practice guidelines to encourage collaboration between multi-disciplinary services results in a highly fragmented system of service providers, and user involvement in the system has been weak (National Mental Health Center of Korea, 2023). Community crisis interventions have been heavily focused on rapid, involuntary hospitalization, leading to high rates of burnout and resignation among mental health workers (Yoon, 2023). The National Human Rights Commission of Korea (NHRCK) reported that human rights protection in the mental health sector in South Korea is weak on several fronts and suggested that the right to self-determination, the provision of options other than hospitalization, and the reduction of coercive treatment are urgently needed (National Human Rights Commission of Korea, 2021).

We can assume that both service users and providers face challenges in South Korea's current system. To overcome this situation, there is a need for new collaboration and dialogue among all stakeholders. The WHO guidelines—which synthesize human rights- and recovery-based approaches proposed in various fields and promote multi-stakeholder, multi-sectoral collaboration—have been suggested as a useful framework for service reform (Cho, 2023).

In 2021, the Ministry of Health and Welfare called for an R&D project to develop training and implementation guidelines for the dissemination of WHO QualityRights-based services in the South Korean context, with requirements to include OD. As part of this project, the first OD introductory workshop in Korea and a subsequent year-long pilot implementation took place. Details of the organization and implementation of the project as a whole and the state of mental health services in Korea in relation to this are described in Supplementary material.

### 1.3 Study objectives

The primary research questions for this study are as follows: (1) *What were the participants' experiences of attending the 5-day OD introductory workshop?* (2) *What were the participants' experiences of 1-year OD pilot practice?* (3) *How did participants' opinions about OD change over the course of 1 year of the OD pilot practice following the workshop as part of the WHO QualityRights Project?*

By addressing these questions, we aimed to gain insights that could provide a basis for designing training and service guidelines to meet the needs of the South Korean mental health system in implementing OD, and also to clarify how OD should reach stakeholders in the field.

The case in this study is unique in that OD was not introduced in isolation but as part of a multi-component service package based on the WHO guidelines. An extended question is therefore to explore the impact of embedding OD within a new human rights-based framework.

## 2 Methods

### 2.1 Study contexts

The R&D projects mentioned above aim to develop OD and non-coercive treatments, supported decision-making, and recovery programs in parallel, and this research focused on the development of OD implementation guidelines and was conducted in the following phases.

#### 2.1.1 Introductory workshop

In March 2023, two international trainers from Finland and the United Kingdom were invited to conduct an introductory workshop for five consecutive days (40 h in total). A total of 28 participants from collaborating organizations participated, including psychiatrists, nurses, social workers, clinical psychologists, peer supporters, family members, and an anthropologist.

The workshop incorporated theories on the seven core principles and 12 key elements of OD, small-group exercises to practice techniques, role-play, and discussions on how to introduce OD.

#### 2.1.2 One-year pilot network meetings

Pilot network meetings have been held on a community basis in Suwon City, the catchment area, since October 2022. During the referral process, a community mental health center promoted the pilot, and an individual or family member called the center and was connected with a team of two to four facilitators for a meeting at their preferred location (most often their home). Clients with suspected or confirmed psychotic symptoms were eligible.

Over the course of the study, 89 network meetings were held with 11 families, with all meetings lasting at least 90 min. The team of facilitators included a Korean psychiatrist (SK) who had completed 1 year of formal OD foundation training and was undergoing international trainer's training in the UK during this period. He attended almost all the sessions to promote fidelity to the key OD elements.

#### 2.1.3 Supervision

Consent was obtained from the pilot clients. Using the video recordings, the researcher (SK) visited London to receive group supervision from international OD trainers. Supervision feedback was shared with colleagues in South Korea.

Monthly supervision meetings were held separately, to which all workshop participants were invited. Further, the project researchers met weekly, during which supervision of the pilot occurred on an *ad-hoc* basis. All meetings were facilitated by the same researcher (SK).

### 2.2 Participants

We selected participants through purposive sampling during an introductory OD workshop in South Korea. A total of 28 people attended the workshops. Of these, 25 consented to participate in the study and completed the first survey. In the second round, 24 of the 25 surveys were returned.

At the end of the pilot year, we categorized this group into those who had experienced network meetings for pilot practice and those who had not.

^*^Network meeting experienced group: Ten people in total who participated in network meetings during the pilot as co-facilitators with a specific researcher (SK) who had undergone formal foundation training and trainer's training.

^*^Network meeting inexperienced group: The remaining group of participants, excluding the above group.

### 2.3 Materials

Participants were provided with a questionnaire booklet that included items to collect demographic data such as gender, age, occupation, and experience with mental health services.

To assess the participants' views of OD and their experiences with the OD workshop and pilot practice, we created an Open Dialogue Opinion Questionnaire based on the questionnaire developed by Razzaque and Wood (2015). The first survey was administered 1 week after the workshop, and the second survey was conducted 1 year later.

The questionnaire was divided into two sections, including qualitative and quantitative elements: one with Likert-type questions and the other with open-ended questions. The Likert-type questions asked participants to rate the seven core principles of OD, as follows (Seikkula et al., 2001, 2003): (1) the provision of immediate help; (2) a social network perspective; (3) flexibility and mobility; (4) responsibility; (5) psychological continuity; (6) tolerance of uncertainty; and (7) dialogism.

For each core principle, participants were asked two Likert-type questions: “To what extent do you agree that each core principle is important in caring for the person?” and “To what extent do you agree that these principles are currently applicable in mental health services in South Korea?” Participants were asked to rate their responses on a 10-point Likert scale ranging from 0 (*strongly disagree*) to 9 (*strongly agree*). Additionally, only in the second questionnaire, participants were asked the following: “How much of each principle do you think you can immediately apply to your workplace?” They were asked to respond using the same Likert-type scale, ranging from 0 (*not currently applicable*) to 9 (*currently applicable*).

Four open-ended questions were asked to obtain participants' qualitative feedback on OD:

- What do you think is important about Open Dialogue?
- What are your opinions about Open Dialogue?
- What challenges do you anticipate in implementing Open Dialogue? (first survey); What challenges have you experienced in implementing Open Dialogue? (second survey)
- How would you explain Open Dialogue to someone who is unfamiliar with it?

Participants were asked two additional questions in the first survey: “What did you like about the Open Dialogue introductory workshop?” and “What did you dislike about the Open Dialogue introductory workshop?” and one additional question only in the second survey: “What support or resources do you think you need to implement and sustain the core principles of Open Dialogue that you rated as highly applicable right now in your workplace?”

### 2.4 Data collection

Participants were informed that the two questionnaires would be sent to their email addresses on the last day of the workshop. Before providing the two questionnaires, participants completed a demographic form and signed a consent form. The first survey was sent after the workshop in March 2023 and collected within 1 month, and the second was sent 1 year later and collected over a month. If the response to an open-ended question was unclear, the first author (SC) contacted the participant via email or text message for clarification to ensure accurate representation.

### 2.5 Ethical considerations

This study was approved by the Institutional Review Board of Ajou University Hospital (AJOUIRB-SB-2023-173). Participants gave written informed consent, were informed about the voluntary nature of their participation, and could withdraw at any time without consequences. The research adhered to national laws and institutional regulations. Data were protected, ensuring anonymity and minimal demographic collection, and stored securely on a password-protected laptop.

### 2.6 Analysis

#### 2.6.1 Variables and statistical analysis

We tested the normality of the survey data using the Shapiro–Wilk test. Student's *t*-test compared age and career length data between groups practicing and not practicing network meetings, while the chi-square test compared other demographic data. For Likert data, we analyzed the mean and standard deviation of scores for the importance and availability of the seven key principles of OD at both time points (*n* = 24). To assess the statistical significance of changes in scores over time for all participants, we conducted paired *t*-tests. Additionally, to evaluate whether the changes in scores over time differed between the group that had experience with network meetings (*n* = 10) and the group that had no experience with network meetings (*n* = 14), we performed a mixed ANOVA to test for interaction effects between time and group experience. Analyses were performed using SPSS version 25.0

#### 2.6.2 Thematic analysis

Data analysis followed (Braun and Clarke, 2006) six-step thematic analysis method. Two authors (SC & SK) immersed themselves in the data, reviewing participants' responses to open-ended questions to identify key semantic units. They independently coded the data, then refined the codes collaboratively, resulting in 750 codes. SC categorized themes by clustering similar codes and delineating overarching narratives, which were reviewed and refined for coherence and relevance. A multi-author validation process involving YHC, a psychiatrist working in a community mental health center, and SKJ, a psychiatrist in hospital services, both of whom participated in the OD introductory workshop, was then undertaken to define and name the themes. This approach provided a more practice-relevant perspective on the data and ensured that the findings accurately reflected real-world contexts. Through consensus and discussion, themes were selected to best encapsulate participants' perspectives, enriched by input from multiple authors. Four main themes and ten subthemes emerged from the analytical process.

## 3 Results

### 3.1 Demographic characteristics

Twenty-four participants completed both the first and second surveys. We included a wide range of people, including psychiatrists, nurses, social workers, clinical psychologists, art therapists, peer supporters, and family activists. We distinguished between those working in hospital settings within the national health insurance system and those working in community organizations funded by the public health budget. The demographic characteristics of all participants are shown in Table 1, and the characteristics of the two groups according to whether they practiced network meetings are shown in Supplementary Table 1.

**Table 1:** Demographic characteristics of participants.

**Demographic**			***N***	***%***	**Mean**	**S.D**.
Age (yr)			24		47.88	10.67
Gender	Male		5	20.8		
	Female		19	79.2		
Occupation	Medical	Psychiatrist	3	12.5		
			Nurse	11	45.8		
	Non-medical	Social worker	4	16.7		
			Psychologist	1	4.2		
			Art therapist	1	4.2		
			Peer support	2	8.3		
			Family activist	2	8.3		
Length of career (yr)			24		16.18	7.31

### 3.2 OD likert-type scale

A Paired *t*-test was conducted to evaluate whether there were statistically significant differences over time in the perceptions of the overall participants regarding the importance and availability of the seven key principles of OD at two time points (t1 and t2) (Table 2). Immediately after the workshop (t1), the mean importance scores for all principles were above 7.71, with dialogism scoring the highest at 8.50. Although availability also scored highest in dialogism, the average was 6.83, and all mean values were observed to be lower compared to their importance scores. One year later (t2), while there was a trend of decreased mean values in importance across all principles, these changes were not statistically significant. In terms of availability, a decline was also observed compared to t1, with flexibility and mobility showing a statistically significant decrease from a mean of 5.79–4.79 (*p* = 0.044).

**Table 2:** Results of a paired *T*-test for measures 1 month post-workshop (t1) and 1 year post-workshop (t2) for all participants.

**Variable**		**Mean (±S.D.)**	***t***	***p***
**Importance**				
Provision of immediate help	t1	7.71 ± 1.65	1.813	0.083
	t2	7.33 ± 1.97		
A social network perspective	t1	8.17 ± 1.07	2.076	0.050
	t2	7.39 ± 1.85		
Flexibility and mobility	t1	7.96 ± 0.95	0.768	0.450
	t2	7.71 ± 1.49		
Responsibility	t1	7.96 ± 0.86	1.440	0.163
	t2	7.50 ± 1.50		
Psychological continuity	t1	7.92 ± 0.97	1.551	0.135
	t2	7.50 ± 1.69		
Tolerance of uncertainty	t1	8.04 ± 1.40	1.440	0.163
	t2	7.58 ± 1.32		
Dialogism	t1	8.50 ± 0.98	0.440	0.664
	t2	8.42 ± 0.83		
**Availability**				
Provision of immediate help	t1	4.63 ± 2.04	0.000	1.000
	t2	4.63 ± 2.39		
A social network perspective	t1	5.92 ± 1.86	0.730	0.478
	t2	5.54 ± 1.82		
Flexibility and mobility	t1	5.79 ± 1.82	2.127^*^	0.044
	t2	4.79 ± 1.59		
Responsibility	t1	5.75 ± 1.70	0.720	0.479
	t2	5.50 ± 1.59		
Psychological continuity	t1	4.29 ± 2.16	−1.496	0.148
	t2	5.13 ± 2.21		
Tolerance of uncertainty	t1	5.17 ± 2.04	−0.920	0.367
	t2	5.54 ± 1.91		
Dialogism	t1	6.83 ± 1.88	0.207	0.838
	t2	6.75 ± 1.54		

NM, network meeting, ^*^p < 0.05.

In the subgroup analysis, participants were divided based on their engagement in network meetings. A mixed ANOVA assessed interaction effects between time (t1 to t2) and group experience, focusing on differences arising from network meeting involvement (Table 3). For importance, both the group that practiced network meetings and the group that did not exhibited similar trends in mean value changes from t1 to t2, with no statistically significant differences. For importance, both the group that practiced network meetings and the group that did not exhibited similar trends in mean value changes from t1 to t2. However, there were no statistically significant differences in the main effect of group (practices NM vs. non-practiced NM), the main effect of time (t1 vs. t2), or the interaction effect between group and time. Conversely, for availability, while there were no significant main effects for group or time on tolerance of uncertainty, a statistically significant interaction effect between group and time was found (*p* = 0.04).

**Table 3:** Results of a mixed ANOVA for measures taken at 1 month post-workshop (t1) and 1 year post-workshop (t2) for participants grouped by network meeting practice.

**Variable**		**Group that practiced NM**	**Group that did not practice NM**	***F***	***p***
		**Mean**	**Mean**		
		**(**±**S.D.)**	**(**±**S.D.)**		
**Importance**					
Provision of immediate help	t1	7.40 ± 1.35	7.93 ± 1.86	0.50	0.49
	t2	7.20 ± 1.99	7.43 ± 2.03		
A social network perspective	t1	8.44 ± 0.88	8.00 ± 1.18	0.06	0.81
	t2	7.78 ± 1.79	7.14 ± 1.92		
Flexibility and mobility	t1	7.90 ± 1.10	8.00 ± 0.88	0.41	0.53
	t2	7.90 ± 0.99	7.57 ± 1.79		
Responsibility	t1	7.80 ± 0.79	8.07 ± 0.92	1.51	0.23
	t2	7.80 ± 1.23	7.29 ± 1.68		
Psychological continuity	t1	7.80 ± 1.03	8.00 ± 0.96	0.003	0.96
	t2	7.40 ± 1.35	7.57 ± 1.95		
Tolerance of uncertainty	t1	8.20 ± 1.03	7.93 ± 1.64	0.90	0.35
	t2	8.10 ± 0.88	7.21 ± 1.48		
Dialogism	t1	8.80 ± 0.42	8.29 ± 1.20	0.01	0.94
	t2	8.70 ± 0.48	8.21 ± 0.97		
**Availability**					
Provision of immediate help	t1	4.80 ± 2.04	4.50 ± 2.10	2.47	0.13
	t2	3.80 ± 2.53	5.21 ± 2.19		
A social network perspective	t1	6.60 ± 1.35	5.43 ± 2.06	0.74	0.40
	t2	5.70 ± 1.70	5.43 ± 1.95		
Flexibility and mobility	t1	5.90 ± 1.73	5.71 ± 1.94	0.28	0.6
	t2	5.20 ± 1.23	4.50 ± 1.79		
Responsibility	t1	5.70 ± 1.49	5.79 ± 1.89	0.13	0.72
	t2	5.60 ± 1.43	5.43 ± 1.74		
Psychological continuity	t1	3.50 ± 2.12	4.86 ± 2.07	0.73	0.40
	t2	4.90 ± 2.28	5.29 ± 2.23		
Tolerance of uncertainty	t1	5.90 ± 1.66	4.64 ± 2.17	4.76	0.04^*^
	t2	5.30 ± 1.83	5.71 ± 2.02		
Dialogism	t1	7.30 ± 1.34	6.50 ± 2.18	0.20	0.66
	t2	7.00 ± 1.76	6.57 ± 1.40		

NM, network meeting, ^*^p < 0.05.

The values of F and p are the values of (group^*^time).

### 3.3 Qualitative results

Four main themes and ten subthemes emerged (Table 4). The main themes and subthemes are summarized below, including representative quotes.

**Table 4:** Main themes and subthemes extracted from the thematic analysis.

**Main theme**	**Sub theme**	**Content**
Uncomfortable ambivalence toward OD: systemic, cultural, and professional challenges in korea vs. human rights potentials	Reluctance and doubt about a different approach to traditional practice	•Systemic challenges •Cultural challenges •Anxiety of new professional roles
	The weighty responsibility of OD implementation as a human rights potential	• Recognizing the limitations of conventional psychiatric services in South Korea • The potentials of OD for human rights restoration
From ambivalence to hope: creating safe spaces with experiential learning of OD in workshop	Gradual immersion in OD through exercises and role-plays	• Healing experiences of being heard and having responses • Recognizing the need for multiple perspectives through reflection exercises • Experiencing different roles and understanding each other
	Creating a safe space and solidarity	• Hierarchy dissolution and individual spontaneity unleashed • Internal ambivalence and diverse external perspectives evolve into polyphony • Building a sense of Solidarity
	Fueled with hope for implementing OD in practice	• Understanding OD as a way of life beyond a mere skill and discovering resources as a facilitator • Expecting OD implementation in various settings and attunement among services
Striving to implement OD as a human rights approach in various settings	Restoring dignity	• Attentive listening and respecting voices • Respecting pace and embracing uncertainty
	The role of Open Dialogue in supporting human rights	• Restoring autonomy and self-determination • Reducing coercion • Promoting collaboration within network and inclusion in community
Identifying challenges and exploring complements for OD implementation	Personal perspectives	• Confusion surrounding the comprehension of OD • Facilitator self-reflection • Maintaining connectivity and sustaining reflective supervision
	Organizational perspectives	• Difficulty in implementing in hierarchical institutional cultures • Creating a new institutional culture
	Policy and institutional perspectives	• Time commitment • Difficulty in ensuring psychological continuity • Safety and legal concerns in crisis intervention • Need for training programs • Policy and institutional support

#### 3.3.1 Main Theme 1. Uncomfortable ambivalence toward OD: systemic, cultural, and professional challenges in Korea vs. human rights potentials

Main Theme 1 illustrates the participants' uncomfortable ambivalence when they first encountered OD in the introductory workshop because it differed from the traditional model. Initially, participants were reluctant and doubtful about implementing OD, perceiving it as challenging to apply in Korea and unsuitable for the Korean context. Despite these reservations, they recognized the need for OD to restore human rights. As workshop participants, they felt the weighty responsibility to gain relevant skills and implement OD in their practice. The subthemes included “Reluctance and Doubt about a Different Approach” and “The Weighty Responsibility of OD Implementation as a Human Rights Potential.”

##### 3.3.1.1 Subtheme 1: Reluctance and doubt about a different approach to traditional practice

Subtheme 1 highlights the reluctance and doubt workshop participants felt about implementing OD domestically, focusing on systemic, cultural, and professional challenges. On the first day of the workshop, participants were introduced to OD's core principles. Many unfamiliar with OD found it markedly different from existing practices, expressing significant concerns about its feasibility in Korea with phrases such as “doubtful,” “uncomfortable,” and “quite challenging” were common (P8, 10, 11, 13, 17, 18, 19, 21).

◦ *Systemic challenges*

Participants identified several systemic challenges to applying OD in Korea, due to differences between the Finnish and Korean healthcare systems (P2, 6, 15, 20, 22). Korea's national health insurance system operates on a fee-for-service basis, and participants questioned the feasibility of integrating OD into this model (P20). Concerns included the lack of specific billing codes for OD (P6, 15, 20), potential funding difficulties (P20), and the risk of OD becoming an exclusive, high-cost treatment (P17, 22).

Additionally, the lack of Korean policies and clinical guidelines supporting OD services was seen as a significant barrier (P9, 13, 24). The fragmented nature of Korea's mental health service delivery system and the absence of guidelines for collaboration between psychiatric hospitals and community services hinder the implementation of OD principles such as “responsibility” and “psychological continuity” (P2, 3, 6, 17, 24).

Participants also highlighted issues related to understaffing and excessive workloads (P1, 2, 3, 5, 6, 7, 10, 11, 14, 16, 17, 19, 20, 21, 22, 25). Frequent job changes and high turnover make it difficult to maintain psychological continuity (P6, 7, 15). Implementing OD while maintaining existing services was seen as difficult due to insufficient numbers of mental health professionals (P11, 21, 25). One participant noted that a single professional in a community mental health center manages over 50–60 clients, in addition to other mandatory tasks (P21). Another highlighted that one psychiatrist in a psychiatric hospital has 60–70 inpatients (P22). Managers expressed reluctance to propose OD because their teams are understaffed and overworked, fearing resentment from staff and pressure from government performance requirements (P8, 12, 16).

◦ *Cultural challenges*

Participants expressed concern that cultural factors in Korea would hinder the application of OD (P3, 4, 7, 8, 12, 16, 20, 22). Specifically, Korea's “*Pali-Pali (hurry-hurry)*” culture contrasts with OD's principles of tolerating uncertainty (P7, 8, 20, 22). This cultural tendency, driven by a sense of urgency, has facilitated rapid economic growth but reflects discomfort with uncertainty (Park, 2019). One participant noted that the “*Pali-Pali”* culture leads service users and families to seek quick solutions, making it challenging to tolerate uncertainty in immediate medical care (P8, 20, 22). They also anticipated related challenges within Korea's rapid healthcare system, where patients can easily access immediate appointments with psychiatrists and receive prescriptions (P20, 22).

Additionally, there was concern that dialogism would be difficult to adopt in a culture where “evaluation and judgment are familiar and silence is considered a virtue” (P2, 3). The emphasis on silence in Korean culture (Robertson, 2019), stemming from “*Nunchi*”—the practice of reading others' feelings and adapting behavior to maintain harmony—contrasts with dialogism.

◦ *Anxiety of new professional roles*

Participants were unfamiliar with the professional roles required in OD and worried that it would take a long time for professionals, clients, and networks to understand and trust OD (P2, 11, 18, 16). They expressed concern about getting clients and networks, especially those in crisis, to understand OD's philosophy, as these individuals often expect quick symptom relief, typically through medication (P2).

While participants were theoretically aware that OD requires professionals to have the courage to embrace new approaches (P2), they found it challenging to let go of the conventional tendency to solve problems (P18). They felt ambivalent about adopting new roles, for “fear of feeling stuck and suffering from low self-esteem” (P17). They found it challenging to implement dialogical attitudes, such as “changing their language,” “being non-judgmental,” and “tolerating uncertainty” (P2, 3, 16).

Participants felt uncomfortable stepping out of their assigned roles within the expert-centered system, with one family activist (P4) expressing fear about facilitating network meetings due to a lack of medical knowledge. Another participant (P9), a peer supporter, doubted her suitability as a facilitator due to a perceived lack of expertise. While one psychiatrist (P13) argued for the active involvement of psychiatrists for comprehensive understanding of clients, another family activist (P3) felt that the absence of a psychiatrist would be limiting.

##### 3.3.1.2 Subtheme 2: The weighty responsibility of OD implementation as a human rights potential

Subtheme 2 describes the responsibility that workshop participants felt toward OD at the beginning of the workshop. Despite recognizing the significant challenges of applying OD in the Korean context, participants understood the potential and necessity of OD to complement conventional mental health services that have human rights limitations. This realization led to a strong, albeit burdensome, sense of responsibility for implementing OD, given its potential to enhance human rights.

◦ *Recognizing the limitations of conventional psychiatric services in South Korea*

While the human rights limitations of mental health services in South Korea were not directly discussed in the workshop, many participants described negative experiences with traditional services. The workshop prompted them to “reconsider the realities and limitations of the traditional medical model” (P11, 22).

Participants recounted the trauma of forced treatment, noting that hospitalization and medication were the default responses to crises (P16, 17, 24). This involuntary treatment led to lifelong psychological trauma (P13, 15, 24), left clients feeling stigmatized and anxious (P5), and caused family conflict and isolation (P1). Clients often lost their social roles and positions after involuntary admission (P6, 14).

The one-way communication typical of traditional psychiatric services was seen as exacerbating client isolation. Providers, “accustomed to authoritative and controlling interventions” (P13), would “systematize clients unilaterally” (P3), “hold therapy meetings exclusively among providers” (P5), and exclude clients from conversations (P5, 14). This approach led clients to become passive and resistant (P13), with “providers burdened by the increased responsibility due to dependence from clients” (P11, 21, 23).

◦ *The potentials of OD for human rights restoration*

In contrast to traditional mental health services, participants found OD to be highly meaningful for realizing clients' human rights values (P5, 13, 14, 23, 25). OD was seen as restoring clients' human rights by giving them agency and control over their psychiatric treatment decisions and fostering mutual accountability (P6, 15, 17, 18, 22, 23, 24). OD was perceived as a “collaborative service” with clients rather than a monopoly of professionals (P13). By returning the initiative to clients, professionals hoped to alleviate their psychological burden and pressure, and to reduce the overwhelming sense of responsibility they felt in traditional mental health services (P5, 11, 13, 15, 20, 21, 24).

#### 3.3.2 Main Theme 2. From ambivalence to hope: creating safe spaces with experiential learning of OD in workshop

Main Theme 2 discusses how participants' initial ambivalence shifted to hope for OD practice through their experience in the OD workshop. Through experiential learning, participants realized that OD is not just a skill but an attitude and a way of life, discovering its practical possibilities. The creation of a safe space allowed participants to voice their internal ambivalence, leading to a natural coexistence of diverse internal and external perspectives. Strong emotional exchanges fostered a sense of solidarity, with participants looking forward to shaping the future of mental health services and implementing OD in their settings. The subthemes were “Gradual Immersion in OD through Exercises and Role-plays,” “Creating a Safe Space and Solidarity,” and “Fueled with Hope for Implementing OD in Practice.”

##### 3.3.2.1 Subtheme 1: Gradual immersion in OD through exercises and role-plays

Subtheme 1 describes how participants were immersed in OD through the workshop's exercises and role-plays. Many participants found these activities to be the most satisfying part of the OD workshop, feeling as though they were participating in real network meetings (P13, 23). One participant noted, “The exercises and role-plays made me realize the significance of OD, which was difficult to accept in theory” (P17).

◦ *Healing experiences of being heard and having responses*

Through these exercises and role-plays, participants had the opportunity to fully share their stories and receive responses, experiencing unconditional listening. Many reported this as a healing experience (P8, 9, 10, 16, 17, 18, 20). A psychiatrist noted, “There are very few opportunities for mental health professionals to share their deepest stories and experience empathy, and this workshop provided a healing experience in a safe space. This experience will help us listen to our clients' stories” (P20).

The following quote is a survey response from a participant who is a peer support worker. She found healing and satisfaction in expressing her deepest feelings during the workshop.

When I went to the doctor... I didn't tell him about my difficulties because I was afraid, he would increase my medication... From the second day of the workshop, I was thinking a lot and crying, but I was able to talk about my feelings and get empathy and listen to other people's stories... I really liked the process. (P9)

◦ *Recognizing the need for multiple perspectives through reflection exercises*

Through reflection exercises, participants acknowledged the value of diverse perspectives in network meetings (P6, 8, 14, 15, 21, 22). They described “how reflection enabled them to hear various inner voices” (P22), “organize their thoughts” (P14), and “gain deeper insight into clients' experiences” (P6). One participant (P8) recognized “reflection as a powerful tool that could deepen understanding and bring unique energy to both clients and network members.”

◦ *Experiencing different roles and understanding each other*

Workshop participants gained a deeper understanding of others by experiencing multiple roles in role-play. After playing the role of the person at the center of concern, one family activist shared, “I thought long and hard about the fact that I could be in the other person's shoes and that our souls are as clear and transparent as a crystal ball when we role-play and connect with each other” (P3). Mental health professionals (P21, 22) found role-plays beneficial in understanding their clients, whereas peer supporters (P9, 14) found it meaningful to play the role of a professional.

##### 3.3.2.2 Subtheme 2: Creating a safe space and solidarity

Subtheme 2 addresses the creation of a safe space, which was crucial in transforming uncomfortable ambivalence into hope. Through experiential learning, participants learned to respect and listen to each other's voices, moving away from perceiving disagreements as requiring argument or persuasion. This process transformed internal ambivalence and external disagreement into polyphony, fostering a safe space where the active exchange of feelings and opinions evolved into a sense of solidarity.

◦ *Hierarchy dissolution and individual spontaneity unleashed*

Despite the short duration of the workshop, the participants experienced significant internal changes and established a safe space together. Initially, there was an imbalance of voices due to an invisible hierarchy among the participants. However, by the last day of the workshop, this hierarchy gradually dissolved, and “everyone felt comfortable engaging in dialogue regardless of rank or status” (P16). Once a safe space was established, “dialogue became more active, and participants' spontaneity emerged” (P24). The following quote is a participant's response that illustrates the change process of incrementally breaking down hierarchies and creating safe spaces:

The youngest participant, who had no clinical experience in psychiatry, became increasingly relaxed, open, and did not care about *Nunchi* as the day progressed. It was touching to see how enthusiastically the other participants responded to her. (P2)

◦ *Internal ambivalence and diverse external perspectives evolve into polyphony*

Once a safe space was created, participants began to freely share their diverse views and perspectives. Their inner ambivalence became an opportunity to “recognize their own desires” (P19) and change their thinking (P6). Participants' voices were no longer about persuasion but about enriching the discussion by engaging with professionals from different organizations, service users, and families (P2, 6, 11, 12, 17, 21, 22). The following quotes illustrate how the voices of different participants created an external and internal polyphony:

It was true polyphony, and I especially appreciated hearing the skeptical perspective on OD during the discussions. Some people asked questions I had been thinking about and shared concerns I hadn't even considered. Before the workshop, my mind was confused and complicated, but after the workshop, I felt a sense of clarity (P22).

◦ *Building a sense of solidarity*

The experience of freely exchanging opinions and feelings in a safe space created a bond between participants (P20) and allowed them to comfort and support each other (P13, 21, 23). One participant reflected on the phrase “people are hope” (P16) and felt they had found “colleagues to share a new paradigm with” (P19, 22). Despite anticipating challenges in securing and practicing OD values in Korea, participants pledged solidarity by remembering the “value of togetherness” (P16) and committing to “trust in the power of the group and process” (P2).

##### 3.3.2.3 Subtheme 3: Fueled with hope for implementing OD in practice

Subtheme 3 captures participants actively planning how they will practice OD after the workshop. On the last day, participants dedicated time to future planning. One participant noted the activeness and proactivity during this process (P24). Participants reflected on their roles as OD facilitators and the value of OD. They returned to their workplaces with concrete plans for OD practice, looking forward to future exchanges and collaborations with workshop resources.

◦ *Understanding OD as a way of life beyond a mere skill and discovering resources as a facilitator*

Initially, participants saw OD as an ideal technique for advancing clients' human rights and felt burdened by the obligation to implement it perfectly. After the workshop, however, they understood OD as a way of life, not just a technique.

The workshops allowed participants to examine their attitudes toward clients and their own lives (P1, 2, 6, 7, 8, 10, 12, 15, 16, 25). They realized that “judging and evaluating others was not conducive to recovery” (P15) and that simply listening could be very helpful (P1, 25). Participants questioned whether they were having authentic dialogue with themselves and others (P8) and were reminded of their own life philosophies and values (P12). They came to see OD as something “more profound than just a therapeutic technique” (P22).

◦ *Expecting OD implementation in various settings and attunement among services*

The workshop process gave participants hope that OD could be applied in Korea (P9, 16). After the workshop, they considered how to implement OD in their workplaces (P2, 4, 8, 11, 16, 17, 24). Participants found it meaningful to gather staff from hospitals and community mental health centers in one space; they expected that OD would be implemented, especially given the focus on person-centered services, with the hope that hospitals and community centers participating in the workshop would collaborate more effectively, even under a fragmented system (P2, 22, 24).

#### 3.3.3 Main Theme 3. Striving to implement OD as a human rights approach in various settings

Main theme 3 reflects the workshop participants' efforts to implement OD in their workplaces. Participants practiced OD in various ways. Ten participants were involved in the network meetings as part of a team of facilitators (P2, 7, 8, 12, 15, 17, 19, 20, 22, 24), as categorized as Network meeting experienced group. Network meeting inexperienced group's participants also tried a dialogical approach at their workplaces by organizing meetings of clients, family members, and professionals gathered in a psychiatric unit (P2, 7, 15, 19, 20, 22), a day hospital (P5, 25), a community mental health center (P1, 12), a suicide prevention center (P18, 21). They applied some principles and elements of OD into their interactions with clients (P1, 3, 6, 7, 15, 18) and used these to self-help groups of service users' organizations (P3, 14). All of them were invited to monthly supervision to share OD practices. Two subthemes emerged: “Restoring Dignity” and “Discovering OD as a Support for Human Rights.”

##### 3.3.3.1 Subtheme 1: Restoring dignity

This subtheme describes how participants used careful listening and patience to move at the client's pace and ultimately work to restore the client's dignity. From their experiences in a variety of settings, participants recognized their importance in OD practice of respecting the client's voice and valuing their journey.

◦ *Attentive listening and respecting voices*

Participants recognized the importance of “fully listening to the client's painful experiences and supporting them in choosing their own path” (P2). They viewed listening to a person's life as a core value of OD (P2, 3, 10) and believed that engaging with the suppressed voice unfiltered (P3) through OD would help clients feel respected (P5, 18).

Focusing on one individual's story for an extended period was a challenge for facilitators (P8, 15, 17). However, they acknowledged the power of authentic listening to drive dialogue. For example, one participant (P2) recalled listening to a client who took more than 10 min to say a single sentence, and eventually witnessing the client feel comforted and open up (P19). And “respecting the voices of all participants in network meetings was seen as crucial for healing” (P15).

Other participants also practiced attentive listening in their own settings. One participant (P7) working in a closed ward described how her initial negative reaction to a client who was self-harming changed after the treatment team used a dialogic approach in which they listened to the client together. Professionals in a day hospital (P5, 25) organized meetings of families and clients in crisis of considering hospitalization and to listen to their struggles and difficulties.

A family activist (P3) changed the way multifamily self-help groups held meetings to a dialogue style, believing that the experience of listening and being listened to would be effective in recovery. A peer supporter (P14) stated that when she facilitates a self-help group, she tries to “honor a variety of voices, including those of the more psychotic, rather than confronting or excluding them.”

◦ *Respecting pace and embracing uncertainty*

Participants recognized that respecting the client's pace and embracing uncertainty are core values of OD. However, pacing was challenging, especially when families had difficulty accepting uncertainty (P19). Families often prioritized solutions over conversation and demanded quick decisions from professionals (P2, 22). Participants empathized with families' impatience and frustration because “they were used to being the answer-givers” (P5). One participant described experiencing “mental burnout from slow change” and wondered in her mind if hospitalization would be a quicker solution (P19).

Gradually, the participants became more comfortable with uncertainty, as did the clients and their families (P17). They found that “the most impressive part of OD is that time moves around the person” (P17) and realized that change requires waiting and that “time has to build up” (P15, 17, 22).

A hospital social worker initially believed that perfect planning and implementation were necessary for change, but she became more accepting of client diversity after practicing the principle of tolerance of uncertainty (P6). A participant from a suicide prevention center (P18) described how waiting for a client's silence led to a trusting relationship: “For a client who was difficult to interview because he was almost nonverbal due to his symptoms, I said, ‘It's okay if you don't say anything right now, you can just be with me for this time,' and I wasn't afraid to wait for his silence. After that, I felt there was trust between us.”

##### 3.3.3.2 Subtheme 2: The role of Open Dialogue in supporting human rights

In subtheme 2, participants noted significant changes in clients and networks through “Attentive Listening and Respecting Voices” and “Respecting Pace and Embracing Uncertainty.” They recognized that OD is a means to protect and facilitate human rights. OD enabled clients to exercise autonomy and self-determination, reducing coercive treatment. It also fostered community inclusion, helping clients find their place within the community. Participants saw OD as a significant example of human rights promotion through positive changes in clients and networks.

◦ *Restoring autonomy and self-determination*

In practicing OD, participants recognized that the professional's role is to respect and facilitate the client's right to self-determination (P5, 7, 15, 20). In particular, they felt that “asking questions that give the client the freedom to choose the time, space, and people they want to meet with is the first key to ensuring their initiative and self-determination” (P14). In fact, they observed that clients and networks felt safer by choosing their own meeting place (P7).

Participants noticed that clients gradually became more self-directed with each session of the network meetings (P17, 22), and one client chose not to take psychiatric medication but continued to voluntarily attend the day hospital program (P2, 17, 19). Eventually, participants realized that “treatment plans that reflected the needs of the client and family reduced dropout” (P6).

Participants previously perceived clients as vulnerable and passive, with limited options (P4); however, during OD practice, participants came to recognize clients as independent beings (P3, 7) who could actively participate in and shape their own destinies (P15) and sought to build a dialogue to ensure that all services were agreed upon (P4).

Through the experience of clients and networks regaining autonomy and self-determination, participants realized that the OD approach is a “recovery system that helps clients and networks understand and choose what they want” (P4) and serves as “a pathway to bring a ‘person-centered', ‘service user perspective to clinical practice”' (P21).

◦ *Reducing coercion*

As participants practice OD in their settings, they have seen OD play an important role in reducing coercive interventions.

One participant attended several network meetings and has witnessed cases where the meetings alone have saved a crisis (P15). A situation that would have resulted in immediate hospitalization by the police in conventional mental health services was resolved through OD (P4). The following quote is a participant's description of a crisis that was resolved through a network meeting.

One client had conflicts with the downstairs neighbor and even called the police, claiming there was the smell of a dead body from the upstairs apartment. Honestly, if they hadn't had the network meetings, I think it wouldn't have been long before they were forcibly hospitalized by the police... In the case of another client, he called his mother and said every night, 'It's really tough because people are stalking me. I'm worried I might hurt someone because of it.' However, almost a year has passed without any forced measures, and now he visits the outpatient clinic on his own and even attends the day hospital. (P15)

Witnessing these cases made it clear to many participants that OD is “a way of working that does not physically or psychologically harm clients in the way that traditional approaches do” (P13, 15, 17, 20, 21, 22, 24). In this sense, one participant defined OD as “a kind, gentle approach” (P2).

Participants in the “Network meeting inexperienced group” who did not participate as facilitators in the network meetings also practiced the values of OD in their workplaces and found it to be a more human rights-consistent approach for clients and families.

One participant (P12) from a community mental health center tried a different approach to intervening with clients in psychiatric emergencies. In South Korea, the Crisis Intervention Team and the police have traditionally conducted rapid emergency hospitalization together, but the participant tried a dialogical approach by bringing together the client's family, the police, social workers from the community center, and mental health professionals prior to hospitalization. As a result, the client voluntarily visited an outpatient clinic and decided to be admitted on his own, which the participant described as “a difficult process that took three times longer than usual, but as a result, I experienced a human rights-centered hospitalization process and became aware of my role as a professional.”

Staff at a university day hospital (P5, 25) saw hospitalizations deferred after holding a family-client dialogue meeting and realized that “even a small amount of communication within the client's network could prevent a forced hospitalization” (P25). A nurse (P7) working in an acute psychiatric unit reported that they had previously used forceful injections and seclusion for patients with challenging behavior, but that they now attempted to have dialogue to understand the psychological factors underlying the patient's behavior before deciding on forceful measures.

The experiences described above resonated with the participants, as they had often witnessed in their work in the mental health field clients being coerced into treatment in crisis situations, resulting in lifelong psychological trauma (P15). For participants, OD was “an opportunity to give a voice to the disempowered” (P2) and “the best option to reduce forced hospitalization” (P20).

◦ *Promoting collaboration within network and inclusion in community*

As participants witnessed the increased collaboration and communication between clients and families, and inclusion within the community, through the OD approach, they came to see OD as “a safe and practical way” to help clients in crisis stay out of the hospital and live as contributing members of society (P2, 6).

Through their experiences of network meetings, participants realized that the process of network and client learning about each other's thoughts and perspectives through dialogue is an important factor in facilitating change (P9, 16), especially “when a large number of members come together to support and empathize with each other, which helps the client's recovery” (P15).

After the network meeting, families modeled the facilitator's conversational style, of listening to the client and understanding their grief, which facilitated communication within the families (P22). This resulted in a gradual change in the way family members treated the client and a change in their attitude toward each other to be more patient (P15). Families also began to take care of themselves, such as voluntarily attending psychiatric clinics to recognize and heal their own minds (P2).

These changes led to positive outcomes in terms of community inclusion, as clients who were reluctant to go outside began to visit art museums with their families (P15), some attended the day center consistently, some got jobs (P2), and some went back to school (P25).

#### 3.3.4 Main Theme 4. Identifying challenges and exploring complements for OD implementation

As participants applied OD in their professional environments, they examined challenges at the personal, institutional, and policy levels, seeking practical solutions. Participants focused on macro-level challenges when faced with OD, as described in Main Theme 1, the main theme 4 highlights participants' growing willingness to identify practical complements for domestic OD practice. This shift indicates that OD is moving beyond theory to concrete human rights practice. The subthemes were Personal, Organizational, and Policy Perspectives.

##### 3.3.4.1 Subtheme 1: Personal perspectives

Subtheme 1 addresses the personal challenges and empowerment regarding practicing OD. Participants experienced confusion in understanding OD concepts and principles, especially in connecting the philosophy to their practice. To address this, they emphasized self-reflection, maintaining connections, and engaging in reflective supervision.

◦ *Confusion surrounding the comprehension of OD*

Participants felt confused about understanding and applying OD. They struggled with multiple internal concerns, such as the worry that OD might merely be a very gentle way to steer clients toward hospitalization and medication, which they wondered was contrary to OD's values (P2, 17). Communicating OD's meaning and practicing listening in peer support groups was also challenging (P3, 14). One participant expressed that OD, not being presented as a manualized theory, could be subjectively interpreted, which might cause confusion (P3).

◦ *Facilitator self-reflection*

To overcome confusion, participants emphasized the importance of self-reflection and mindfulness in their role as facilitators (P2, 8, 16).

◦ *Maintaining connectivity and sustaining reflective supervision*

Participants highlighted the importance of supervision in practicing OD (P2, 3, 6, 14, 15, 16). Ongoing supervision ensures the exchange of ideas and growth (P14, 15, 16), preventing network meetings from becoming “for-profit time-filling programs” (P2). Effective team chemistry is crucial, and regular meetings should foster relationships among team members (P6). Participants also emphasized the need to share and make sense of the confusion (P2, 3).

##### 3.3.4.2 Subtheme 2: Organizational perspectives

Subtheme 2 presents the challenges and strengths of practicing OD from an institutional perspective.

◦ *Difficulty in implementing in hierarchical institutional cultures*

Participants found organizing network meetings within hierarchical healthcare organizations challenging. One participant (P22) explained that although her organization was founded on the principles of the therapeutic community, it was a hospital where the main goal was to relieve patients' symptoms; therefore, vertical communication was prioritized. The first meeting was organized in a top-down manner by a manager. As a result, expressing opinions on an equal footing while facilitating with her boss was challenging, impacting teamwork and hindering the ability to tolerate uncertainty during meetings (P2, 20). Suggestions for improvement were often disregarded. They also faced role confusion and resistance from service users and families to the new approach, which impacted the effectiveness of meetings (P2, 20, 22).

◦ *Creating a new institutional culture*

Several participants emphasized the need for a receptive and collaborative culture to successfully implement OD (P2, 5, 6, 18, 19, 22, 24). They highlighted “the importance of feeling connected to coworkers and growing together” (P18), “fostering an atmosphere that embraces a recovery perspective” (P22), and “striving to connect people with their communities” (P5).

##### 3.3.4.3 Subtheme 3: Policy and institutional perspectives

Subtheme 3 describes the policy and institutional challenges participants faced in practicing OD and suggests solutions. Key issues included time commitment, ensuring psychological continuity within a fragmented mental health system, safety and legal issues in crisis intervention, training of professionals, and the need for institutional support.

◦ *Time commitment*

Participants worked extra hours to practice OD while maintaining their existing jobs, leading to increased overtime and psychological distress (P12, 15, 17, 18, 19, 20, 22, 25). Participants who took part in the network meeting highlighted the challenges of dedicating half of their workday to traveling to a client's home and facilitating the meeting (P2, 15, 19, 20, 22).

◦ *Difficulty in ensuring psychological continuity*

The fragmented mental health system in Korea makes it challenging to ensure psychological continuity in OD practice. For example, a network meeting was interrupted due to a lack of cooperation when a client was suddenly hospitalized (P22, 24). This highlighted “the need for a system that links patients from hospitalization to discharge” (P6, 25).

◦ *Safety and legal concerns in crisis intervention*

Participants expressed concerns about safety, liability, and lack of legal protection when applying OD in psychiatric emergencies (P1, 7, 20). There were questions about whether OD could be used effectively in suicide crises (P21) and the role of facilitators in emergencies (P14).

◦ *Need for training programs*

Many participants emphasized the need to train professionals to spread OD in Korea (P3, 4, 6, 7, 9, 15, 17, 19, 20, 22, 25). They mentioned the importance of an organization to operate and train people around OD, ensuring high-quality education and training (P4, 9, 20).

◦ *Policy and institutional support*

Participants stressed the need for institutional support and supply chains to enable OD access (P3, 7, 15, 19, 20, 21, 22, 24). They suggested policy support to embed training and supervision into basic work (P24), financial support and practice guidelines to promote a recovery perspective (P3, 20, 22, 24), and additional charges for staff (P7, 15). Qualitative evaluation methods, given OD's nature, and legal protection for facilitators in crisis situations were also recommended (P20, 21).

## 4 Discussion

The aim of this study is to explore the subjective experiences and opinions of participants involved in a 5-day OD introductory workshop and 1-year pilot practice as part of the WHO QualityRights Project in South Korea. According to the qualitative results, participants initially felt ambivalent toward OD due to systemic, cultural, and professional challenges in Korea, which led to reluctance and doubt. However, they also recognized its human rights potential and felt the weighty responsibility to implement OD (Main Theme 1). By the end of the workshop, their ambivalence had shifted to hope through experiential learning, fostering solidarity and optimism for future OD practice (Main Theme 2). After the workshop, participants implemented OD by restoring clients' dignity and autonomy, which reduced coercion and increased community inclusion (Main theme 3). They also identified and addressed personal, organizational, and policy challenges in practicing OD (Main theme 4). This study could provide foundational data for developing a formal training program and implementation guidelines for OD in the Korean mental health system.

The quantitative analysis employed two methods: a paired *t*-test for the entire participant group and a mixed ANOVA based on network meeting experience. The paired *t*-test revealed that, among the seven key principles, only “Flexibility and Mobility” in terms of availability showed a statistically significant decrease over time. This result may reflect the structure of mental health care system in South Korea, which is characterized by fragmentation and a provider-centered approach, limiting the flexibility required to meet the individual needs of clients. Furthermore, the finding could have been influenced by the fact that participants involved in network meetings reported feeling burdened by the time and effort required to travel to the client's home (Main Theme 4, Subtheme 3: Time Commitment).

Second, the mixed ANOVA results indicated a statistically significant decline in the availability of Tolerance of Uncertainty among participants who engaged in network meetings over the course of 1 year. Although tolerance of uncertainty is a key principle of OD, participants faced considerable challenges in sustaining it during network meetings (Main Theme 3, Subtheme 1). Factors such as rapid conclusions, traditional interventions, hypotheses, and assessment tools were found to obstruct the cultivation of tolerance for uncertainty and hinder the creation of a trustworthy therapeutic context or “scene” (Seikkula and Olson, 2003). The qualitative analysis suggests that participants were employed in institutions that predominantly relied on these conventional practices, which may have further complicated their efforts to maintain tolerance for uncertainty. This may explain the observed decline in its availability in the quantitative analysis. These findings align with prior research, which has highlighted similar difficulties faced by OD practitioners working within Treatment as Usual (TAU) environments when attempting to implement OD (Anestis et al., 2024). Fostering tolerance for uncertainty requires teamwork, and successful co-therapy necessitates creating space for both verbal and physical attunement (e.g., mindfulness) and for maintaining relationships (e.g., supervision) (Lagogianni et al., 2023). This is consistent with Main Theme 4, as identified by participants in this study.

Additionally, it is notable that “Dialogism” consistently scored the highest on both Likert-type scales assessing importance, availability, and immediate applicability, as measured in the survey at both points in time. The implications of this result will be discussed in the qualitative analysis that follows. This suggests that participants experienced OD, quite literally, through open dialogue at its core, both during the workshop and in the 1-year pilot practice.

In discussing the qualitative results, we will examine the implications across three key areas: training, practice, and team/policy dynamics.

### 4.1 Training

As with most studies examining the opinions and experiences of professionals in countries that first adopted OD, participants expressed reluctance and suspicion toward OD, citing numerous challenges to its initial adoption. The implementation of OD may “generate organizational, professional, and personal resistances,” leading to significant challenges in its acceptance and adoption (Weber and Johansen, 2007; Søndergaard, 2010). In the UK, a survey of professionals before OD's introduction indicated resistance, considering OD in the NHS as a radical shift (Razzaque and Wood, 2015). Initial impressions of OD have been described as fearful and threatening, with concerns about changing professional roles (Razzaque and Wood, 2015), anxiety over incompetence and criticism in Greece (Skourteli et al., 2023), and ongoing resistance management at clinical and organizational levels in Australian private hospitals (Lennon et al., 2023).

A novel finding of this study is the ambivalence, not just resistance, professionals feel toward OD. Similar ambivalence was noted in Australian private healthcare, where professionals were both optimistic and skeptical during OD training and implementation (Dawson et al., 2021).

Ambivalence, as defined by attitudinal ambivalence, involves conflicting positive and negative feelings about the same object, prompting efforts to resolve these conflicts (Jonas and Ziegler, 2007). This state of ambivalence is perceived as highly uncomfortable, leading individuals to actively seek ways to resolve the conflict between incompatible evaluations (Newby-Clark et al., 2002). Addressing ambivalence is crucial when introducing or training for OD. Specifically, applying the key factors identified in this study that influence the transformation from ambivalence to hope in the training process may assist future trainees in managing their ambivalence more effectively when planning and facilitating future OD trainings.

This study highlights that the weighty responsibility felt by participants is a crucial factor that needs to be addressed to facilitate OD training. Participants viewed OD as an ideal method for advancing clients' human rights but felt burdened by the obligation to implement it flawlessly. Stockmann et al. (2019) reported that some OD trainees in the multi-center ODDESSI trial found it challenging to practice OD within “a system prioritizing a technical approach.” This suggests that treating OD as a skill to be perfected can be burdensome.

From a psychodynamic understanding of mental illness, the power imbalance between service providers and users is often explained by unconscious processes that lead to a role-assignment in which the professional assumes a “only healthy, knowledgeable, kind, powerful, and active” position and the patient assumes a “only ill, suffering, ignorant, passive, obedient, and grateful” position (Hinshelwood, 2004, p. 14). Since the not-knowing stance emphasized in OD contradicts this, professionals who need to be perceived as knowledgeable may struggle to accept “the courage to be vulnerable” (Lorenz-Artz et al., 2023) during the OD process, or they might feel overwhelmed, treating OD like a new psychotherapy technique to be mastered.

The findings suggest that it is important to facilitate the experience of OD as a way of life rather than a method to be practiced during training. The debate on whether OD should be viewed as “psychotherapy” or “a way of life” has been ongoing (Ong et al., 2019). Seikkula (2011) describes dialogism as a “way of life” learned through communication from birth. Simply listening, responding, and exchanging responses—elements that are already embodied from early childhood experiences—can be healing. It can be hypothesized that participants re-experiencing these fundamental elements during the workshop helped them embrace OD as a way of life, giving them confidence in their practice.

We identified two factors crucial for transforming ambivalence and weighty responsibility into hope: the content of the training and the organization of the training course.

#### 4.1.1 Training content

Although this study involved a short, 5-day workshop, the results were consistent with participants' experiences in longer training courses in several aspects. Participants in a 3-year training course experienced unexpected healing, reporting changes in the co-production of meaning, language, and relationships due to a climate of trust (Runciman, 2021). In a 1-year foundation training, participants felt responded to and listened to through exercises and role-plays, gaining insights into the emotions of clients and network members in crisis (Aderhold and Hohn, 2021; Hendry et al., 2021). Similarly, a 4-day introductory training showed that participants adapted to dialogical practice and experienced inner knowing (Thorley et al., 2023).

Therefore, the findings of this study suggest that improving the potential impact of short-term OD workshops require placing less emphasis on the introduction of OD principles or theories, and more on experiential learning and “bodily knowing of OD” (Shotter, 2007) about OD as a way of life and the nature of processes. Through experiential learning, participants gradually became accustomed to dialogical practice, no longer perceiving initial ambivalence as something to be dealt with. They were able to exist in polyphony rather than seeing differing opinions as needing persuasion or unification. While not confirmed in this study, it is possible that this was achieved by the trainer creating a safe dialogical space for different voices and encouraging polyphony. Although the participants did not mention specifically trainers' intervention, this could indicate that trainers very naturally facilitated a dialogical culture by participating as containers within the dialogical space (Thorley et al., 2023). This gentle process may have made participants feel as though they were learning autonomously.

Additionally, organizing the training to prioritize experiential learning over merely explaining OD principles helps participants understand OD as a way of life. A qualitative study found that participating in a network meeting was the most authentic way to grasp OD, rather than first explaining its principles (Lorenz-Artz et al., 2023).

#### 4.1.2 Strengths of mixed participant populations

In this study, the workshops brought together professionals, peer supporters, and family members from various community organizations and healthcare facilities in the catchment area where OD was being introduced. This mixed-group trainee structure was adapted from the QualityRights training tool (World Health Organization, 2019), which encourages mixed groups with participants from different backgrounds (professionals, service users). The mixed-group structure mirrors that of OD's network meetings. Participants in this study felt a sense of solidarity and hope through the workshops, a finding confirmed in other studies. In a 3-year OD training course in the UK, the training group itself practiced interactive ways of accepting differences of opinion, tolerating difficult emotions, and overcoming internal tensions during discussions (Wates et al., 2022). In an OD training in Australia, participants felt a strong sense of connection among themselves and learned by joining others (Buus et al., 2022). In a POD training, participants felt an emotional connection with people (Stockmann et al., 2019).

Thus, when planning and organizing short term OD training, it can be suggested that including professionals, peer supporters, and family members from local organizations and healthcare facilities in the catchment area where OD is to be introduced can foster a sense of solidarity and hope for future OD practice.

Moreover, the word “hope” appears several times in the participants' reports. This finding can be explained by the suggestion that hope is a shared practice rather than a personal sentiment and that it operates as a kind of language (Cuffari et al., 2022).

**Figure 1:**
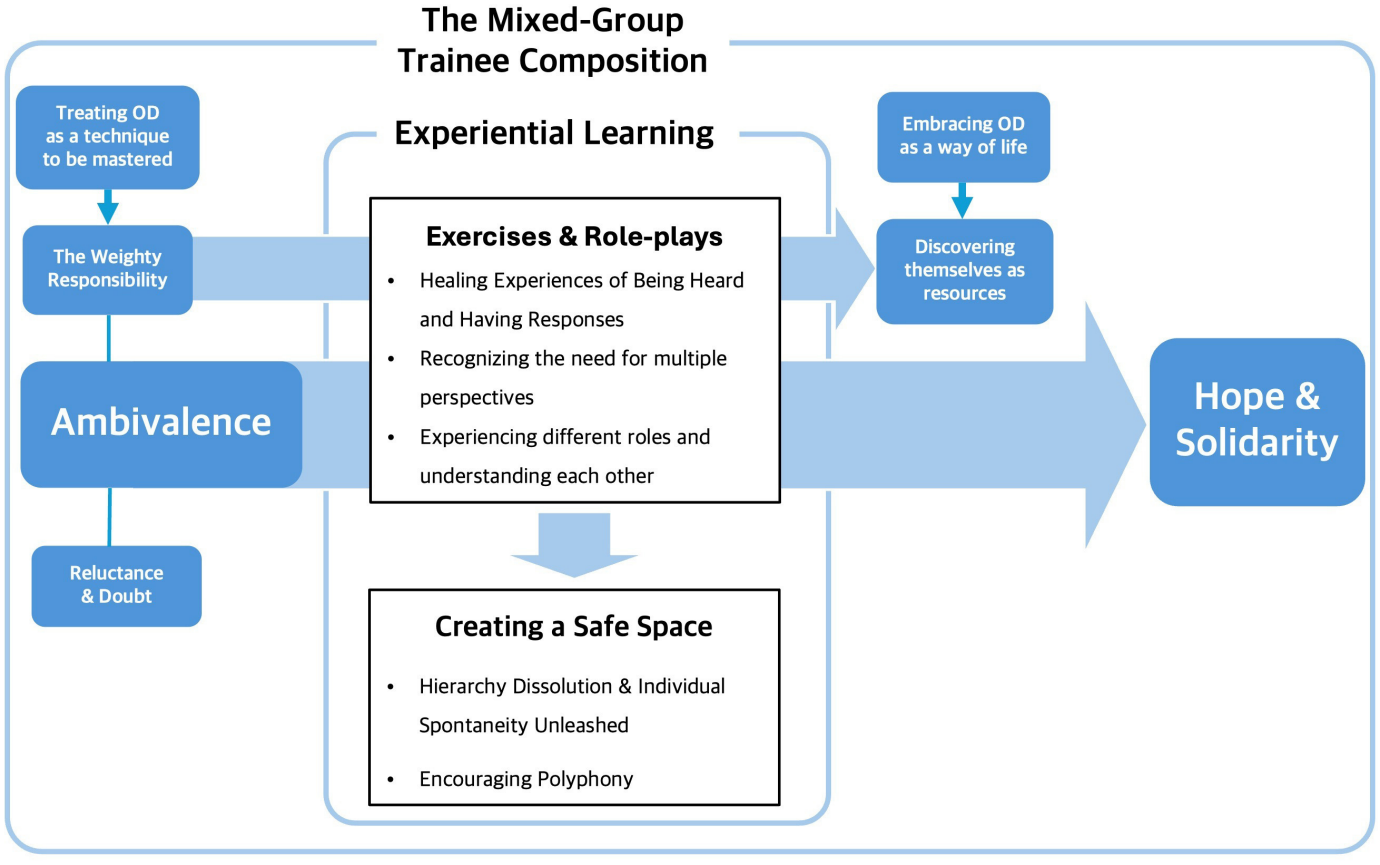
The process of change from ambivalence to hope and solidarity during the OD workshop.

Figure 1 illustrates the process of the change from participants' initial ambivalence to hope and solidarity for OD practice during the introductory workshop. Initially, the ambivalence did not manifest as conflict or argumentation but rather transformed into solidarity and hope. This change can be attributed to the qualitative impact of experiential learning, the content of the training, and the strength of the mixed group. The collective hope and solidarity fostered by OD motivated participants to embrace a new approach throughout the year.

### 4.2 Practice: OD as a human rights-aligned approach

Adopting a human rights perspective was useful in formulating the meaning-making of Main Theme 3. Despite human rights being a global concern in the context of mental health, little research has explored the direct relevance of OD to human rights principles. It has been suggested that OD should be considered a human rights-aligned approach, as many elements of the CRPD that underpin QualityRights are consistent with the fundamental principles of OD (Von Peter et al., 2019), and the WHO's guidance outlines the value of OD from a human rights perspective (WHO, 2021). Therefore, the findings reported in this article suggest that the practice of OD can contribute to securing the human rights of clients and networks.

Initially, participants in this study strived to honor the voices and respect the pace of their clients and network members through attentive listening. As a result, they facilitated self-determination and autonomy. The study results underscore that OD practice aligns with the principles of the CRPD, specifically Article 21, which asserts the right to freedom of expression and opinion, and Article 3, which emphasizes respect for inherent dignity, individual autonomy, including the freedom to make one's own choices, and independence of persons. Clients' experiences of regaining dignity, autonomy, and self-determination through OD have been documented in several studies and are consistent with the findings of this study. For instance, Sidis et al. (2020) reported that young clients felt empowered to say what they wanted during network meetings. Similarly, clients who experienced OD in the UK valued the experience of having a choice and voice, being involved in treatment planning, and discussing their mental health needs above all other themes (Sunthararajah et al., 2022). Additionally, the WHO suggests OD as a model of supported decision-making that respects the will of mental health service users (World Health Organization, 2019, p. 28).

Moreover, Von Peter et al. (2019) suggested that future research should examine how OD affects different forms of coercion. Encouragingly, our study found that OD can indeed prevent various forms of coercion. This is compatible with CRPD's the Article 14: Liberty and Security of Person, ensuring that persons with disabilities are not deprived of their liberty unlawfully or arbitrarily; Article 15: Freedom from Torture or Cruel, Inhuman, or Degrading Treatment or Punishment; and Article 16: Freedom from Exploitation, Violence, and Abuse.

Considering the existing literature, one professional who implemented an approach developed by adapting OD in Vermont, USA, described OD as less exhausting and more humane because it does not involve taking away people's freedom or autonomy (Florence et al., 2020). Furthermore, OD is featured in the Council of Europe's compendium of good practices aimed at eliminating coercive practices in mental health settings as a matter of human rights (Gooding, 2021).

Finally, participants in this study observed that practicing OD facilitated the client's inclusion into their network and community by working collaboratively with them. These findings suggest that OD can be considered to have a significant human rights impact, aligning with Article 4 of the CRPD, which emphasizes general obligations to closely consult with and actively involve persons with disabilities in the development and implementation of legislation and policies. For instance, a UK client described how OD network meetings helped him reconnect with his mother, creating a ripple effect that supported his reintegration into the community (Hodgkins and Debra, 2021). This narrative underscores the potential of OD to foster meaningful relationships and support systems that uphold the dignity and rights of individuals.

Furthermore, these observations highlight the broader implications of OD as a method that not only addresses immediate mental health needs but also promotes long-term social integration and community participation.

### 4.3 Teams/policies

In Main Theme 4, where challenges and compliments were mentioned at multiple levels, participants suggested personal reflection, supervision, and connections within the team as solutions to overcome the confusion associated with OD implementation. This opinion juxtaposes the suggestion that ongoing and effective supervision is crucial for sustaining OD (Jacobsen et al., 2023). This is also consistent with the suggestion that trust among team members is a prerequisite for OD practices, which require mutual acceptance and attunement (Lagogianni et al., 2023).

In qualitative findings, this opinion moves into the need for organizational culture change. There are reports that network meetings within hospitals have been difficult to implement because of the hospital's hierarchical culture and innate goal of alleviating symptoms. This is consistent with the opinion that integrating OD into existing treatment settings can be challenging due to differences in underlying assumptions and values (Ong et al., 2019), and with the opinion that it may be even more difficult in psychiatric clinical settings where academic theories and expert models are applied to individual suffering (Schütze, 2021).

However, there are also studies that have shown positive effects when OD is applied in a modified form in a hospital setting (Rosen and Stoklosa, 2016; Ritva et al., 2018); therefore, it is also proposed that OD should be considered in a form that is tailored to the circumstances of the institution (Heumann et al., 2023), and that even if only some aspects of OD are introduced, there is value in doing so (Schütze, 2021). In order to shift the culture of care in this direction, it has been suggested that organizational and leadership-level changes are required, particularly by cultivating cultural change and adaptation and by continually removing organizational obstacles, which can be done by holding the anxieties and frustrations of different parts of the organization (Lennon et al., 2023). In order to achieve this organizational change, the criteria (Olson, 2021) for organizations that want to adopt OD can be a significant reference.

At a higher level, there were many comments about the need for policies and budgets for OD to be established; the results of this study present a policy proposal, and it is necessary to include policy guidelines that address this need. For example, the same qualitative findings from this study— that OD practices can result in time-consuming overtime and confusing legal liability—are echoed in other studies (Heumann et al., 2023).

Other qualitative comments about the need for formal training, guidance to maintain psychological continuity under fragmented services, and funding for sustainable implementation also suggest the need for policy change at multiple levels. The example of the UK ODDESSI trial (Razzaque, 2021), where training and implementation are conducted within the context of a large national research platform, can be an important reference. Further, the top-down implementation in Italy (Macario et al., 2021; Pocobello et al., 2024), driven by eight mental health departments, can also serve as a reference for policy design.

These multilevel qualitative findings resonate with the suggestion (Aarons et al., 2011) to consider the individual, organizational, and system levels in policy planning. In the context of South Korea, with the aforementioned recommendations of the National Human Rights Commission (National Human Rights Commission of Korea, 2021) and the inclusion of WHO QualityRights in the new Mental Health Policy Innovation Plan (Kim, 2023), the R&D project, including this study, has the potential to become a new platform for OD to be implemented. QualityRights is similar to OD and participatory in that it involves all stakeholders—professionals, service users, and families—in a collaborative way (World Health Organization, 2019), and has been shown to be effective in improving service quality and human rights when applied to systems in a region (Pathare et al., 2021).

The significant emergence of human rights-related subthemes in main theme 3 of the qualitative findings may be related to the fact that this study was not an OD training alone but was combined with other QualityRights trainings such as Non-Coercive Treatment and Supported Decision Making. We can also assume that the mixed-stakeholder trainee group setting recommended by QualityRights contributed to the extraordinary sense of solidarity in this workshop. This points to the potential for complementarities between WHO QualityRights and OD and suggests the need for further research.

## 5 Strength and limitation

This study has several strengths. Firstly, the longitudinal design allowed for the observation of changes in participants' experiences and opinions regarding OD practice over a year following the workshop. This provides valuable insights into long-term impact of OD practices. Secondly, to maintain adherence to core OD principles and elements, the author (SK), with formal training and trainers' training, led the pilot practice under the supervision of international experts, promoting the fidelity of the OD practice. Lastly, few studies have explored the relationship between OD and human rights. The study highlights its potential as a human rights-aligned approach, emphasizing its importance in mental health services.

However, the study also has some limitations. Firstly, the small number of participants (*n*) limits the interpretation of quantitative results. Future studies should include larger sample sizes to enhance statistical power and generalizability. Secondly, the institutions involved in this study had a strong culture of recovery practice, which may not reflect typical South Korean institutions. Participants' familiarity with human rights principles might have influenced the outcomes. Thirdly, not all participants practiced OD, and the study includes relatively few opinions from those who did not implement the approach, making it difficult to understand their barriers to practicing OD. Future research should focus on these participants to gain insights into the challenges faced. Lastly, due to resource constraints, interviews were not conducted. Although participants provided detailed responses to open-ended questions, future studies should incorporate interviews to obtain more in-depth results.

## 6 Conclusion

The conclusion of this preliminary study regarding the formal introduction of OD as part of the WHO QualityRights service package in South Korea can be summarized as follows.

Because the great success of OD in Lapland is considered to be based on high-quality training (Putman, 2021a), full-scale training inside the formal system is necessary to successfully introduce OD. This is preceded by the need to increase social awareness and consensus among stakeholders regarding OD. However, owing to the nature of OD, it is difficult to convey the core principles only through literature or lectures; this potentially leads to confusion or resistance due to misconceptions (Lorenz-Artz et al., 2023).

This study shows that even a short, well-planned, and well-designed introductory workshop can significantly motivate participants unfamiliar with OD and provide clues as to what the key learning agent of the introductory workshop should be.

Empowering and motivating participants through OD workshops has a multifaceted, positive impact not only on OD practices but also on the way participants work as well as on teamwork in traditional settings. Further, from a human rights perspective, these changes can have practical implications that translate values into real service in many ways. In this respect, the study provides new evidence to support OD as a good human rights-based service.

The study could be a new example of OD being disseminated as a top-down policy by a country's R&D projects and also the first case of OD being introduced as part of the WHO QualityRights service package. In this unique context, the study implies that OD and this global human rights-based mental health project have the potential to complement each other.

## Data Availability

The original contributions presented in the study are included in the article/Supplementary material, further inquiries can be directed to the corresponding author.
