# Supplementary material for: Introducing Open Dialogue as part of the WHO QualityRights Project in South Korea: experiences and opinions from an introductory workshop and 1-year pilot practice
Source: Front Psychol. 2024 Oct 17;15:1426122. doi: 10.3389/fpsyg.2024.1426122 (PMC11525595; doi:10.3389/fpsyg.2024.1426122)
Supplement: Supplementary file 1 [file Table_1.DOCX]

Supplementary Material

Introducing Open Dialogue as Part of the WHO QualityRights Project in South Korea: Experiences and Opinions from Introductory workshop and One-Year Pilot Practice

Sooni Cho^1†^, Yong Hyuk Cho^2†^, Jai Sung Noh^2^, Seong Kwon Jeong^1^, Shin Kwon Kim^3^, and Seongsu Kim^4*^

* Senior & Corresponding author: Seongsu Kim

Email: [mindtrek@hanmail.net](mailto:mindtrek@hanmail.net)

$\dagger$ These authors have contributed equally to this study and share first authorship.

1. **State of mental health services in South Korea and introduction of the WHO QualityRights project**

In South Korea, in terms of mental health crisis interventions and treatment for psychosis, psychiatric treatment is concentrated in inpatient units at hospitals, whereas psychosocial rehabilitation and recovery approaches are offered at community service organizations after discharge. Funding for both comes from the national health insurance system on the one hand and the public health budget on the other. There is a lack of legislation and practice guidelines to encourage collaboration between multi-disciplinary services, resulting in a highly fragmented system of service providers. Additionally, peer support work by service users and family members with lived experiences takes place within mental health services but has not been recognized as a formal service, and user involvement in the system has been weak (National Mental Health Center of Korea, 2023). Community crisis interventions have been heavily focused on rapid, involuntary hospitalization, leading to high rates of burnout and resignation among mental health workers (Yoon, 2023). The National Human Rights Commission of Korea (NHRCK) reported that human rights protection in the mental health sector in South Korea is weak on several fronts and suggested that the right to self-determination, the provision of options other than hospitalization, and the reduction of coercive treatment are urgently needed (National Human Rights Commission of Korea, 2021). We can assume that both service users and providers face challenges in South Korea’s current system. To overcome this situation, there is a need for new collaboration and dialogue among all stakeholders. The WHO guidelines—which synthesize human rights- and recovery-based approaches proposed in various fields and promote multi-stakeholder, multi-sectoral collaboration—can be a useful framework for service reform.

In South Korea, dialogical and recovery-based, multi-disciplinary teamwork in mental health crisis response and acute inpatient care was first applied in a newly opened provincial psychiatric hospital in 2020 (Korea Biomedical review, 2020). The hospital began to offer the services suggested by the WHO QualityRights initiative, including OD-inspired network meetings, non-coercive treatment, recovery planning programs, and supported decision-making from peer supporters, synchronously starting immediately after admission. The network meetings were organized by the hospital’s director, a psychiatrist with formal foundation training in OD, who motivated the staff. After two years of implementation, several key health indicators were significantly better than the Korean average, including readmission rates one month after discharge, the mechanical restraint rate, and linkage to community services, despite a fairly short hospital stay (Kang, 2022).

Since these new attempts have been made, service user organizations in South Korea have consistently called for the adoption of WHO QualityRights-based services and OD, and there is a growing consensus on the existence and need for such human rights-based and person-centered services (Park, 2021). As a result, the need for WHO QualityRights-based services has been mentioned in the recent revision of the Korean Mental Health Act (Cho, 2023) and the new Mental Health Policy Innovation Plan (Kim, 2023); there are also plans for such services to be incorporated into the formalization of future rights-based services (such as peer support, supported decision-making, and non-coercive treatment).

1. **National research and development(R&D) project to develop WHO QualityRights based services including Open Dialogue**

In 2021, the Ministry of Health and Welfare called for an R&D project to develop training and implementation guidelines for the dissemination of WHO QualityRights-based services in the South Korean context, with requirements to include OD. One university and one psychiatric hospital were selected as collaborative research sites; this R&D project is being conducted over a 3-year period from 2022 to 2024. Suwon City was selected as the catchment area. The collaborating organizations include one university hospital, one general hospital with psychiatric wards, one psychiatric hospital, three community mental health centers, two service user organizations, and one family organization.

The project’s development goals are as follows (Cho, 2023):

Section A. Develop training tools or guidance:

1. Open Dialogue-based network meetings to be applied in community services

2. Open Dialogue-based network meetings for hospital services

3. Non-coercive treatment, including de-escalation techniques

4. Supported decision-making by peer supporters for legal capacity support

5. Recovery planning as a multi-stakeholder group practice

Section B. Training workshops for the five components, followed by pilot operations. Based on these experiences, operational guidelines should be developed to ensure that they function in a synchronized manner as a service package within the South Korean mental health system.

Items 3, 4, and 5 of the five components in Section A (as stated above) were developed using the WHO QualityRights face-to-face training tools as a core framework, which was adapted to be actionable in the South Korean system and cultural context. After drafting each tool, face-to-face training workshops were held for each item at various levels. The workshops included multi-disciplinary professionals from collaborative organizations, as well as service users, and family members.

Items 1 and 2 of Section A are related to OD. The process for developing them is described in the methods section of the main article.

Additionally, apart from the pilot network meetings at the community level focused on in the main article, the participating hospitals held their own inpatient network meetings, of which one psychiatric hospital held 62 sessions with 32 families over the course of the study. These meetings were aimed at gaining experience in developing network meetings within hospitals.

As mentioned earlier, those who participated in the OD workshop, the subsequent pilot, and supervision were part of the overall research project collaborators. Therefore, many of them had taken part in other workshops on topics including non-coercive treatment, recovery planning, and supported decision-making.

The ultimate goal of R&D is the development of a full-scale training introduction plan and a proposal for service system change by analyzing cases in other countries, performing a literature review, gathering feedback from participants, and holding public hearings with stakeholders. Given the nature of OD—which does not lend itself to manualization, the outcome will be a source of guidance on structures for implementing and sustaining the service, covering aspects such as training, supervision, research, collaboration with peer supporters, organizational culture, institutional arrangements, and policy frameworks. The present study is also part of such efforts.

1. **References**

Cho, Y. H. (2023). Development and Dissemination of Human Rights Based/Recovery Oriented Mental Health Services in Korea. Available at: https://www.nmhc.or.kr/sub.php?id=nmhc&mode=view&menukey=17&ctgrstr=%EC%A0%95%EC%B1%85%ED%8F%AC%EB%9F%BC&idx=8265&page=1&menukey=17

Kang, S. (2022). Survey to establish a human rights-friendly treatment environment for people with mental disabilities(Korean). National Human Rights Commission of Korea. Available at: https://www.humanrights.go.kr/base/board/read?boardManagementNo=17&boardNo=7608928&searchCategory=&page=1&searchType=total&searchWord=%EC%A0%95%EC%8B%A0&menuLevel=3&menuNo=115

Kim, E. (2023). Will these 4 CounterMeasures Save South Korea from Mental Illness? *KBR*. Available at: https://www.koreabiomed.com/news/articleView.html?idxno=22719 (Accessed April 13, 2024).

Korea Biomedical review (2020). “Crisis response center will change mental health service.” *KBR*. Available at: https://www.koreabiomed.com/news/articleView.html?idxno=8352 (Accessed October 12, 2023).

National Human Rights Commission of Korea (2021). Human Rights Report for People with Mental Disabilities. Available at: https://www.humanrights.go.kr/site/program/board/basicboard/www.humanrights.go.kr (Accessed April 13, 2024).

National Mental Health Center of Korea (2023). National Mental Health Statistics 2021(Korean). Available at: https://www.ncmh.go.kr/ncmh/board/boardView.do?no=9290&fno=106&bn=newsView&menu_cd=04_02_02_04&bno=&pageIndex=1&search_item=&search_content=

Park, J. E. (2021). [Press conference] Human rights treatment stranded..."We support human rights-friendly treatment at the new Gyeonggi Provincial Psychiatric Hospital". Available at: http://www.mindpost.or.kr/news/articleView.html?idxno=5176

Yoon, J. (2023). Employment insecurity, work overload... “Mental health centers” with holes [In-depth feature - Shaking up the national mental health care system]. *Segyeilbo*. Available at: https://www.segye.com/view/20230424514570 (Accessed April 13, 2024).

**Supplementary Table S1.** Demographic characteristics of participants grouped according to whether they practiced Network Meeting.

| **demographic** | | | **Group that**  **practiced NM** | | | | **Group that**  **did not practice NM** | | | |
| --- | --- | --- | --- | --- | --- | --- | --- | --- | --- | --- |
|  |  |  | ***N*** | ***%*** | **Mean** | **S.D.** | ***N*** | ***%*** | **Mean** | **S.D.** |
| Age(yr) | | | 10 |  | 47.40 | 12.37 | 14 |  | 48.21 | 9.76 |
| Gender | Male | | 2 | 80.0 |  |  | 3 | 21.4 |  |  |
|  | Female | | 8 | 20.0 |  |  | 11 | 78.6 |  |  |
| Occupation | medical | Psychiatrist | 2 | 20.0 |  |  | 1 | 7.1 |  |  |
|  |  | Nurse | 6 | 60.0 |  |  | 5 | 35.7 |  |  |
|  | Non-medical | Social worker |  |  |  |  | 4 | 28.6 |  |  |
|  |  | Psychologist | 1 | 10.0 |  |  |  |  |  |  |
|  |  | Art therapist | 1 | 10.0 |  |  |  |  |  |  |
|  |  | Peer Support |  |  |  |  | 2 | 14.3 |  |  |
|  |  | Family Activist |  |  |  |  | 2 | 14.3 |  |  |
| Length of Career(yr) | | | 10 |  | 17.75 | 9.18 | 14 |  | 15.05 | 5.74 |

*NM=Network Meeting*
